# Determinants of a high prevalence of cesarean section among women in eastern Uganda

**DOI:** 10.11604/pamj.2023.46.90.38208

**Published:** 2023-11-23

**Authors:** Alimah Komuhangi, Racheal Akello, Jonathan Izudi

**Affiliations:** 1 Institute of Public Health, Clarke International University, Kampala, Uganda,; 2 Afya na Haki, Gayaza, Nakwero, Uganda,; 3 Department of Community Health, Mbarara University of Science and Technology, Mbarara, Uganda,; 4 Data Science and Evaluations (DSE) Unit, African Population and Health Research Center (APHRC), Nairobi, Kenya

**Keywords:** Postpartum women, caesarean section, Uganda

## Abstract

**Introduction:**

increasing proportion of Uganda women deliver by cesarean section (C-section) but limited studies have examined the determinants of C-section. We investigated the prevalence and determinants of C-section among women aged 15-49 years in eastern Uganda.

**Methods:**

we retrieved data for women who attended postnatal care across four large healthcare facilities in Kamuli district. C-section (surgical operation to deliver a baby through the abdomen, whether planned or not) was the outcome. Binary logistic regression was done to determine factors independently associated with C-section, reported as adjusted odds ratio (aOR) and 95% confidence interval (Cl).

**Results:**

of 727 participants, 126 (17.3%) had delivered by C-section, with the associated factors as self (aOR=1.92, 95% CI 1.04-3.52) and unemployment (aOR=1.81; 95% CI 1.01-3.21), birth order namely second (aOR=3.13, 95% CI 1.77- 5.65), third (aOR=3.60, 95% CI 1.97-6.78), fourth (aOR=2.88, 95% CI 1.46-5.93) and fifth or beyond birth (aOR=2.16, 95% CI, 1.17-4.09), and a rural health facility (aOR=2.04, 95% CI 1.31-3.22).

**Conclusion:**

the C-section prevalence is slightly higher than recommended by the World Health Organization. There is a need to promote contraceptive use to limit fertility, increase access to contraceptives among rural women, raise awareness among women about the importance of early and regular antenatal visits through education campaigns, equip healthcare facilities with well-trained staff and infrastructure to ensure quality antenatal care to prevent complications that could lead to C-sections, and conduct ongoing research to identify barriers and challenges faced by women in seeking quality healthcare and knowledge about obstetric risk factors.

## Introduction

Nearly 75% of all maternal deaths result from five major complications namely, hemorrhage, obstructed labor, sepsis (infection), eclampsia (convulsions), and unsafe abortion, all of which are treatable [1]. The majority of all maternal deaths (94%) are in low and middle-income countries [1]. Estimates suggest that 40% of all pregnancies end up with obstetric complications and will require cesarean delivery or C-section [2], an important component of comprehensive emergency obstetric care [3]. The rate of C-section has risen rapidly over the years in both developed and developing countries [4]. In developing countries such as Uganda, recent data suggest an 8% (47% to 51%) increase in the rate of C-section [5], and the reasons for this increase are not completely understood. Direct causes of maternal death such as postpartum hemorrhage, obstructed labor, and pre-eclampsia among others might be one of the plausible reasons but there are social, economic, cultural, and health systems-related factors as well.

Epidemiological studies show that several factors are associated with C-section in both developed and developing countries. One study revealed urban residence, first delivery after 35 years of age, and fetal malpresentation such as breech presentation [6], availability of health insurance [7], low-income or poverty, and previous history of C-section [8] are a few of the notable factors associated with C-section. The C-section rate in Kamuli district in eastern Uganda stands at 6.6% according to July 2020 to June 2021 data, which is substantially higher than the WHO recommended rate of 5% [9]. The reasons for the higher rate of C-section in the district remain unclear. Accordingly, we investigated the determinants of C-section among women who had received intrapartum care at four large health facilities in the district. This information might help the district in designing strategies to reduce the C-section rate and might be useful in similar settings.

## Methods

### Data source and setting

The data for this study were retrieved from a previous health facility-based cross-sectional study conducted in Kamuli district. Elsewhere [10], the study is described. Kamuli district is found in the east-central region of Uganda and has an estimated total population of 545,900 people according to the latest survey [11]. The parent study enrolled 875 participants of which 148 had delivered at home. The study established the prevalence and factors associated with pre-lacteal feeding among postpartum women in Kamuli district [10] and was conducted at four large healthcare facilities. The study population included postpartum women aged 15-49 years sampled from four health facilities, namely Nankandulo Health Center IV, Namwendwa Health Center IV, Kamuli General Hospital, and Kamuli Mission Hospital. The first three health facilities are government-owned while the last is the only private health facility in the district. These health facilities provide antenatal care, delivery/intrapartum care, and postnatal care services. The health facilities are accessible, operating 24 hours a day and 7 days per week. In the present study, we excluded women who had home delivery because they were not assessed for C-section. The inclusion of these women would result in the wrong estimate of C-section prevalence.

**Study design and ethics approval:** we conducted a secondary analysis of data from a previous study [10]. We reported the findings following the Strengthening of Observational Studies in Epidemiology guidelines [12]. Since the parent study received ethical approval from Clarke International Research Ethics Committee (reference # CLARKE-2020-23), we were granted a waiver of informed consent to analyze the data. Findings of the study are reported according to the Strengthening of the Reporting of Observational Studies in Epidemiology (STROBE) guidelines.

### Measurements

**Outcome variable:** the outcome variable was C-section measured by self-report on a dichotomous scale. Participants were asked the question “at the last pregnancy, by what means did you give birth?” Participants who reported that they had a delivery through the surgical operation of the uterus constituted C-section and all the other modes of delivery, namely spontaneous and assisted vaginal delivery were considered as none C-section cases.

**Independent variables:** the independent variables included maternal factors, namely age measured as absolute years and then categorized as 15-24 years and ≥ 25 years; ethnicity (Basoga, Baganda, Basamia, and Bagishu), level of education (none, primary, secondary and tertiary/university), type of employment (formal, self and none/unemployed), marital status (single/never married, married and divorced/separated), religion (Christian, Muslim and others) and maternal residence (urban, peri-urban and rural). The obstetric factors included birth order (first, second, third, fourth, and fifth and beyond), human immunodeficiency virus (HIV) status (positive or negative), birth weight in kilograms (< 2.5, 2-5-4.0, and ≥4.0), number of ANC visits at the recent pregnancy (< 4 or ≥ 4). The health facility-related factors included level of health facility (health center or general hospital), and access to health facility measured by estimated distance from home to the health facility as ≤ 5km versus > 5km.

**Data analysis:** the analysis was performed in R version 4.0.2 (2020-06-22). We performed exploratory data analysis where we summarized numerical data using mean and standard deviation and categorical data using frequencies and percentages. We conducted a bivariate analysis to assess statistically significant differences in C-section with various factors using the Chi-square or Fisher's exact tests. Variables at the bivariate analysis level with p < 0.15 and those deemed relevant from the literature were included in the multivariable analysis where we fitted a generalized linear model with a logit link and binomial distribution to determine factors independently associated with C-section. Variables that did not improve the model fit as measured by the log-likelihood were dropped to achieve model parsimony. The findings were reported as odds ratios (OR) and 95% confidence interval.

## Results

**General characteristics of study subjects:** Table 1 describes the participant characteristics. Overall, we studied 727 participants with a mean age of 26.23±5.89 years, 408 (56.1%) were aged ≥ 25 years, 534 (76. 2%) were of the Catholic religious faith, 334 (45.9%) attained a secondary level of education, 265 (38.5%) were peri-urban residents, 601 (82.7%) were married, and 355 (48.8%) were unemployed.

**Table 1:** participant characteristics

Characteristics	Categories	Total (n= 727), No. (%)
Age group (years)	15-24	319 (43.9)
	25 and beyond	408 (56.1)
	mean (SD))	26.23 (5.89)
Religion	Catholic	534 (76.2)
	Muslim	158 (22.5)
	Others	9 (1.3)
Level of education	None	60 (8.3)
	Primary	257 (35.4)
	Secondary	334 (45.9)
	Tertiary/university	76 (10.5)
Residence	Urban	182 (25.0)
	Peri-urban	280 (38.5)
	Rural	265 (36.5)
Marital status	Single/never married	89 (12.2)
	Married	601 (82.7)
	Divorced/separated	37 (5.1)
Type of employment	Formal	93 (12.8)
	Self	279 (38.4)
	None	355 (48.8)

### Differences in C-section with maternal, obstetric, and health facility-related factors

The differences in C-section with maternal, obstetric, and health facility-related factors are shown in Table 2. Overall, 126 (17.3%) participants had C-section. C-section was more prevalent among women aged 15-24 years (17.9%), Catholic religious faith (17.8%), women with tertiary/university level of education (26.3%), urban residents (20.9%), and single/or never married (25.8%) among others. We observed statistically significant differences in C-section concerning religion (p = 0.04), marital status (p-value: 0.031), employment status (p = 0.002), birth weight (p < 0. 001), birth order (p < 0. 001), distance to health facility (p= 0.002), and the location of health facility (p<0.001).

**Table 2:** bivariate analysis of differences in C-section with maternal, obstetric, and health facility-related factors

Characteristics	Level	Caesarean delivery (n=126)	Spontaneous vaginal delivery (n=601)	P-value
No. (%)	No. (%)	
Age group (years)	15-24	57 (17.9)	262 (82.1)	0.811
	≥25	69 (16.9)	339 (83.1)	
	mean (SD)	26.2 (7.1)	26.2 (5.6)	0.984
Level of education	None	11 (18.3)	49 (81.7)	0.150
	Primary	44 (17.1)	213 (82.9)	
	Secondary	51 (15.3)	283 (84.7)	
	Tertiary/university	20 (26.3)	56 (73.7)	
Marital status	Single/never married	23 (25.8)	66 (74.2)	0.031
	Married	94 (15.6)	507 (84.4)	
	Divorced/separated	9 (24.3)	28 (75.7)	
Type of employment	Formal	28 (30.1)	65 (69.9)	0.002
	Self	40 (14.3)	239 (85.7)	
	None	58 (16.3)	297 (83.7)	
Ever received health education at ANC	No	38 (21.7)	137 (78.3)	0.100
	Yes	88 (15.9)	464 (84.1)	
Birth weight	<2.5	10 (26.3)	28 (73.7)	<0.001
	2.5-4	85 (14.0)	522 (86.0)	
	≥4	31 (37.8)	51 (62.2)	
Birth order	First	44 (28.8)	109 (71.2)	<0.001
	Second	24 (12.6)	167 (87.4)	
	Third	20 (11.8)	150 (88.2)	
	Fourth	15 (15.8)	80 (84.2)	
	Fifth and beyond	23 (19.5)	95 (80.5)	
Distance to health facility	≤5	63 (13.8)	392 (86.2)	0.002
	>5	63 (23.2)	209 (76.8)	
Location of health facility	Urban	90 (21.6)	327 (78.4)	0.001
	Rural	36 (11.6)	274 (88.4)	
Type of health facility	Private-not-for profit	31 (17.4)	147 (82.6)	1.000
	Government	95 (17.3)	454 (82.7)	

### Factors associated with C-section at the unadjusted and adjusted analysis

In the unadjusted analysis (Table 3), C-section was more likely among the unemployed (OR, 2.21; 95% CI, 1.29- 3.71) and self-employed women (OR, 2.57; 95% CI, 1.47- 4.48), women who had delivered babies with birth weights between 2.5-4.0 kgs (OR, 2.19; 95% CI, 1.98- 4.54), women with the second (OR, 2.81; 95% CI, 1.63- 4.94), third (OR, 3.03; 95% CI, 1.71- 5.52) and fourth birth orders (OR, 2.15; 95% CI, 1.14- 4.25), as well as women who had delivered in a rural health facility (COR, 2.09; 95% CI, 1.39- 3.22). In the adjusted analysis, we excluded distance to the health facility, religion, and marital status because these variables did not improve the model fit. The adjusted analysis showed the following factors were associated with C-section: self-employment (aOR,1.92; 95% CI, 1.04 - 3.52), none employment (aOR,1.81; 95% CI, 1.01-3.21), birth orders namely second (aOR, 3.13; 95% CI, 1.77- 5.65), third (aOR, 3.60; 95% CI, 1.97- 6.78), fourth (aOR, 2.88; 95% CI, 1.46-5.93) and ≥ fifth birth (aOR, 2.16; 95% CI, 1.17- 4.09), and rural health facility location (aOR, 2.04; 95% CI, 1.31-3.22) (Table 3).

**Table 3:** factors associated with C-section

Characteristics	Level	Unadjusted analysis (OR, 95% CI)	Adjusted analysis (aOR, 95% CI)
Religion	Catholic	1	
	Muslim	1.41 (0.86-2.40)	
	Others	0.17* (0.04-0.67)	
Marital status	Single/never married	1	
	Married	1.88* (1.10-3.13)	
	Divorced/separated	1.08 (0.46-2.74)	
Type of employment	Formal	1	
	Self	2.57*** (1.47-4.48)	1.92* (1.04-3.52)
	None	2.21** (1.29-3.71)	1.81* (1.01-3.21)
Birth weight (Kgs)	<2.5	1	1
	2.5-4	2.19* (1.98-4.54)	1.73 (0.76-3.69)
	≥4	0.59 (0.24-1.34)	0.49 (0.19-1.19)
Birth order	First	1	1
	Second	2.81*** (1.63-4.94)	3.13*** (1.77-5.65)
	Third	3.03*** (1.71-5.52)	3.60*** (1.97-6.78)
	Fourth	2.15* (1.14-4.25)	2.88** (1.46-5.93)
	Fifth and beyond	1.67 (0.95-3.00)	2.16* (1.17-4.09)
Distance to a health facility	≤5	1	
	>5	0.53**(0.36-0.79)	
Location of health facility	Urban	1	
	Rural	2.09*** (1.39-3.22)	2.04** (1.31-3.22)

**Note:** significance codes at 5% level: p<0.001***, p<0.01**, p<0.05*.

## Discussion

This study focused on the determinants of C-section in Kamuli district in eastern Uganda. The data show that 17.3% of the women had C-section, which is slightly higher than the prevalence of C-section in a recent study in eastern Uganda which puts the prevalence at 14% [13]. The current prevalence of C-section is twice lower than the estimated national health facility-based C-section prevalence of 36% in 2021 [14] and a 25% prevalence in a previous study in Kabarole district, mid-western Uganda [15]. Our results suggest that, the C-section rate in this setting is higher than the recommended WHO desired estimate of 10-15%. Accordingly, more effort is required in designing strategies to reduce the observed C-section rate.

Our study shows that women without employment and those who are self-employed are more likely to undergo C-section compared to those in formal employment. Our finding disagrees with the results of a previous study [4] which shows employed women are more likely to undergo C-section. The discrepancy in the findings could be attributed to differences in the study setting. In our setting, the women in the formal employment sector largely represent the elite class, who are potentially more knowledgeable about obstetric risk factors for C-section. Such women are more likely to seek quality healthcare services during pregnancy and this might have contributed to early identification and correction of obstetric risk factors hence the reduced chance of C-section. Another probable explanation could be related to working hours. Conversely, women in formal employment have well-defined working hours compared to those without employment or in self-employment and this might have influenced C-section. A previous study by Hung *et al*. (2002) [16] reports longer hours of work as a predisposing factor to C-section. Besides, women without employment are likely poor and poverty has been reported as an important predisposing factor for C-section [17]. This is because poor women tend work for longer hours.

The current study revealed a high likelihood of C-section at rural health facilities than urban health facilities. Our finding can be explained by several factors. Access to health care in rural areas is challenging [18] and women are likely to report to the health facilities with complications that could have been prevented had access been easy, just like in an urban setting. Another plausible explanation could be that rural health facilities largely provide services to rural women who get exposed to the risks of pregnancy at a much younger age through early marriage [19]. In addition, urban facilities are technologically more advanced than rural health facilities, often equipped with ultrasound scans for example and this enables early risk detection and management hence preventing C-section [20]. Rural health facilities do not have such diagnostic capacities [21]. Differences in skills set and experience between healthcare providers at the rural and the urban health facilities might be another factor. In the former, the decisions to perform C-section may be more irrational than the latter [22].

We found that the likelihood of women delivering by C- section increased with the number of births. This finding is consistent with previous studies in Pakistan [23] and sub-Saharan countries [24]. These studies linked pregnancy-related risks to an increased number of pregnancies. Our study setting has a higher fertility rate of 6.8 compared to the national average of 5.8 [25]. The high fertility rate could have potentially predisposed women to pregnancy-related risks requiring C-section.

**Study strengths and limitations:** our study has strengths and limitations. Regarding the strengths, to the best of our knowledge, this study is among the first few studies on determinants of C-section in the study setting and rural eastern Uganda in general. The data analyzed were from health facilities with the highest patient loads in the district so the results are likely generalizable. The limitations include the lack of data concerning obstetric risk factors for C-section such as cephalopelvic disproportion, fetal malposition, fetal malpresentation, pre-eclampsia/eclampsia, and obstructed labour.

## Conclusion

Our study shows that C-section is prevalent among women in Kamuli district and that C-section is more likely among women who are none or self-employed, women with birth order ≥ 2, and among women who sought care at a rural health facility. Our findings suggest a need to promote contraceptive use in order to limit fertility with a specific target on women accessing maternal health care services in rural health facilities and women with none or self-employment. There is a need to raise awareness among women about the importance of early and regular antenatal visits through education campaigns and continuous research, equip healthcare facilities with well-trained staff and infrastructure to ensure quality antenatal care to prevent complications that could lead to C-sections, and conduct ongoing research to identify barriers and challenges faced by women in seeking quality healthcare and knowledge about obstetric risk factors. These data will be useful to continuously refine and improve healthcare delivery and education programs.

### What is known about this topic

- *Maternal mortality ratio is high in low-income counties;*
- *Cesarean section is a life saving intervention for women with obstetric complications;*
- *On average, 10-15% of the women with obstetric complications undergo C/section.*

### What this study adds

- *This is the first study to investigate the level and determinants of on C/section among rural women in eastern Uganda;*
- *Our data show that C/section is more likely in unemployed women than employed women;*
- *The study reveals that rural health facilities perform C/section more than urban health facilities.*
